# Novel LAT Pathogenic Variants in a POI Family and Its Role in the Ovary

**DOI:** 10.3389/fgene.2021.764160

**Published:** 2021-11-19

**Authors:** Kun Chu, Yi He, Ziyuan Li, Zhongxin Jiang, Liang Wang, Yixuan Ji, Xiang Wang, Wenjuan Pang, Ningxia Sun, Fu Yang, Wen Li

**Affiliations:** ^1^ Center of Reproductive Medicine, Shanghai Key Laboratory of Embryo Original Diseases, International Peace Maternity and Child Health Hospital, School of Medicine, Shanghai Jiao Tong University, Shanghai, China; ^2^ Department of Obstetrics and Gynecology, the PLA Rocket Force Characteristic Medical Center, Beijing, China; ^3^ Department of Obstetrics and Gynecology, No. 905 Hospital of PLA Navy, Shanghai, China; ^4^ Center of Reproductive Medicine, Changzheng Hospital, Naval Medical University, Shanghai, China; ^5^ Department of Medical Genetics, Naval Medical University, Shanghai, China

**Keywords:** genetic variants, primary ovarian insufficiency, the linker for the activation of T cells, granulosa cell, whole-exome sequencing, genetic varaiant

## Abstract

Premature ovarian insufficiency (POI) affects about 1% of women under 40 years and leads most often to definitive infertility with adverse health outcomes. Genetic factor has been reported to play an important role in POI. However, the genetic etiology remains unknown in the majority of the POI patients. Whole-exome sequencing and variant analysis were carried out in a POI pedigree. *In vitro* studies of the wild-type and mutant proteins were conducted in primary granulosa cells (GCs) and granulosa cell line. The result showed that the patients carried compound heterozygous nonsynonymous mutations (c.245C > T and c.181C > G) in *LAT* gene, which were identified to be transmitted from their parents. The two variants were assessed to affect residues that were conserved across different species examined, and were predicted to be deleterious by software predictions. Protein structure predicting result indicated that the two variants could alter their interactions with surrounding residues, which may change the internal structure of the LAT protein. Moreover, LAT protein expression in GCs was demonstrated for the first time, and further functional assays suggested that this mutation could reduce LAT expression and influence GC survival, which may contribute to the etiology of POI. In summary, we detect novel *LAT* pathogenic variants in a POI pedigree and report for the first time that LAT is present and functional in the GCs of the ovary. Our findings not only shed new light on the role of LAT in GCs, but also broaden the spectrum of genetic causes of POI.

## 1 Introduction

Premature ovarian insufficiency (POI), also named premature ovarian failure (POF) or premature menopause, is defined as loss of ovarian function before the age of 40. Characterized by amenorrhea, hypoestrogenism, and elevated gonadotropin level, POI often results in compromised infertility, associated with an increased risk of cardiovascular disease, osteoporosis, vulvovaginal atrophy, impaired sexual function, neurological effects, and overall reduced life expectancy (Webber et al., 2016). POI is estimated to affect about 1% women under 40 years, and its pathogenesis is still under-researched (Qin et al., 2015; Tucker et al., 2016).

Apart from the possible environmental and autoimmune factor effects, genetic factor has been reported to play an important role in POI pathogenesis, accounting for approximately 20–25% of the POI cases (Qin et al., 2015). Identifying precise pathogenic genes has been challenging, since the disease is highly genetic heterogeneous, which is associated with numerous genes related to ovarian development and meiosis pathway (Franca et al., 2019). In addition, investigating the genetic architecture of sporadic POI cases remains a difficult task, because of the diverse gene variants and inheritance patterns among individuals.

Low prevalence and impaired fecundity have contributed to limited POI pedigrees, which makes whole-exome sequencing (WES) technology an effective approach to study its causative genes. To date, a large number of genetic mutations have been identified in POI cases, and are reported to be mostly associated with folliculogenesis, such as *BMP15* and *NOBOX* (Di Pasquale et al., 2004; Chand et al., 2006; Qin et al., 2007; Lechowska et al., 2011). Despite the progress we have made in recent years, the genetic etiology remains unknown in the majority of the POI patients.

In this study, we performed WES in a POI pedigree and identified novel compound heterozygous mutations (c.245C > T and c.181C > G) in *LAT* gene. LAT protein expression in granulosa cells (GCs) were demonstrated for the first time. Further *in vitro* functional assays suggested that this mutation could suppress proliferation and promote apoptosis of GCs, which may contribute to the etiology of POI.

## 2 Materials and Methods

### 2.1 Ethics Statement

The Ethics Committee of Changzheng Hospital gave a positive approval for this study. Written consent was obtained from all participants included in the study.

### 2.2 Genetic Analysis

Blood samples of the family pedigree were isolated and preserved in EDTA tubes after obtaining informed written consent. Whole-exome sequencing and variant analysis were conducted by iGeneTech Bioscience Co., Ltd. The PCR products were collected with agarose gel electrophoresis and Purelink PCR Purification Kit (Thermo Fisher Scientific, United States), and further analyzed by directional sequencing according to the manufacturer’s protocol. The amino acid sequence of LAT protein from different species was obtained from Uniprot, and multiple sequence alignments were performed using the Clustal W tool.

### 2.3 Denovo Protein Structure Predicting

Rosetta Software was used to analyze the changes in tertiary structure of mutant LAT protein. The optimized protein model was evaluated by PROCHECK and ERRAT programs. Ramachandran plot was used to describe the rotation degree of the bonds between Cα atoms and carbon atoms (psi), and the bonds between Cα atoms and the nitrogen atoms (phi) in the peptide bonds, which indicates the allowable and disallowed conformations of amino acids. ERRAT is a program for evaluating the three-dimensional structure of a protein based on crystallography. The evaluation function of ERRAT is mainly obtained by considering the non-bond interactions in the crystal structures of proteins, also relating to the resolution of them.

### 2.4 Experiment Animals

Female C57BL/8J mice of 4 weeks, 8 weeks, and 24 weeks old were used in this study. Mouse ovaries were dissected from adherent tissues and collected for experiments. All animal protocols were approved by the Ethics Committee of Changzheng Hospital.

### 2.5 Construction of Expression Plasmids

Wild-type (pENTER-LAT) and mutant (pENTER-mutLAT: P82L, P61A) *LAT* cDNA original from human were constructed. *LAT* cDNA sequence’s information was obtained from NCBI (www.ncbi.nlm.nih.gov), and the vectors containing the mutant *LAT* gene were designed based on the mutant sites of the patients.

### 2.6 Cell Culture and Transfection

COV434 cells were cultured in Dulbecco’s modified Eagle medium (Gibco, United States) supplemented with 10% fetal bovine serum (Thermo scientific, United States), 1% penicillin/streptomycin (Thermo scientific, United States). Wild-type and mutant LAT reconstructed vectors were transfected into COV434 cells with Lipofectamine 3,000 (Thermo scientific, United States) based on the manufacturer’s protocol. For each transfection, 5 μL Lipofectamine 3,000 transfection reagent was added to 100 μL Opti-MEM (Thermo scientific, United States) pre-dissolved with 2 μg template plasmid and incubated for 15 min at room temperature. 70–90% confluent COV434 cells were cultivated with the complex and were harvested at 48 h for analysis.

### 2.7 Western Blotting

Transfected GCs were lysed with RIPA lysis buffer (Beyotime, China) based on the manufacturer’s protocol. Protein concentrations were measured with the BCA Protein Assay kit (Abcam, United States), after which protein samples were separated by 8% SDS-PAGE, transferred to PVDF membranes (Millipore, United States), and blocked in 10% skimmed milk for 1 h at room temperature. Membranes were incubated with primary antibodies overnight at 4 °C, washed 3 times with TBST, and then exposed to secondary antibodies for 2 h at room temperature. Protein bands were detected by Microchemi 4.2 device (Bio-Rad, United States) using an enhanced chemiluminescent (ECL) reagent kit. The related antibodies included anti-LAT (1:1,000, Affinity), HRP labeled anti-β-actin (1:10,000, Proteintech Group) and HRP labeled goat anti-mouse/rabbit IgG (1:3,000, DingGuo Bio).

### 2.8 Immunohistochemistry

Paraffin-embedded mouse ovarian tissue sections were deparaffinized, immersed, heated, blocked, and then incubated overnight with the LAT antibodies (1:100, Abcam, overnight, 4 °C). Localization of the primary antibody was performed by incubation of the sections with the corresponding secondary antibodies (Invitrogen) at 1:500 dilution for 1 h at room temperature. Finally, nuclei were stained with hematoxylin. At least three different samples from each genotype were analyzed in parallel.

### 2.9 Immunofluorescent Staining

Transfected cells were fixed using 2% paraformaldehyde (room temperature, 15 min), washed in PBS (three times), permeabilized (0.1% Triton X-100, 5 min), blocked (3% BSA in PBS, 1 h), and incubated with LAT antibody (1:500, Affinity, overnight, 4 °C). Antibodies binding was detected with Alexa-Fluor-488-conjugated donkey anti-rabbit secondary antibody (1:500, 1 h, RT), followed by treatment with DAPI (1:1,000, 5 min, RT). Confocal images were acquired with the microscope (NIKON Eclipse Ti, Japan), and no less than 20 fields of transfected cells were captured for each sample.

### 2.10 EdU Staining

The proliferation of GC was measured by 5-ethynyl-2-deoxyuridine (EdU) uptake following the manufacturer’s instructions (Click-iT Edu Imaging Kit, Invitrogen). In brief, cultured cells were incubated with EdU for 30 min, and then fixed with 4% PFA at room temperature. Following washes in 3% BSA twice, cells were incubated for 30 min at room temperature in PBS plus 0.5% Triton X-100, washed in 3% BSA twice, and then incubated with the reagents in the Kit.

### 2.11 Flow Cytometry

The apoptosis of GC was measured with Annexin V-APC/7-ADD Apoptosis Analysis Kit (Shanghai Universal Biotech Co., Ltd, China). Cells were washed with PBS and then centrifuged at 1,500 rpm for 3 min. 1.0 x 10^5^ cells were collected and suspended with 500 μL Binding buffer. After 5 μL Annexin V-APC and 5 μL 7-ADD were added, the cell suspension was incubated for 30 min on ice in the dark. FACSJazz (Becton Dickinson) was used for analysis and sorting.

### 2.12 Statistical Analysis

Statistical analyses were performed with SPSS 21.0 and GraphPad Prism (two-tailed Student’s *t* tests or one-way analysis of variance). All data were presented as mean ± standard deviation (SD) from at least three repeats of independent experiment. *p* < 0.05 was considered to be significant.

## 3 Results

### 3.1 Novel LAT Pathogenic Variants Identified in a POI Family

**FIGURE 1:**
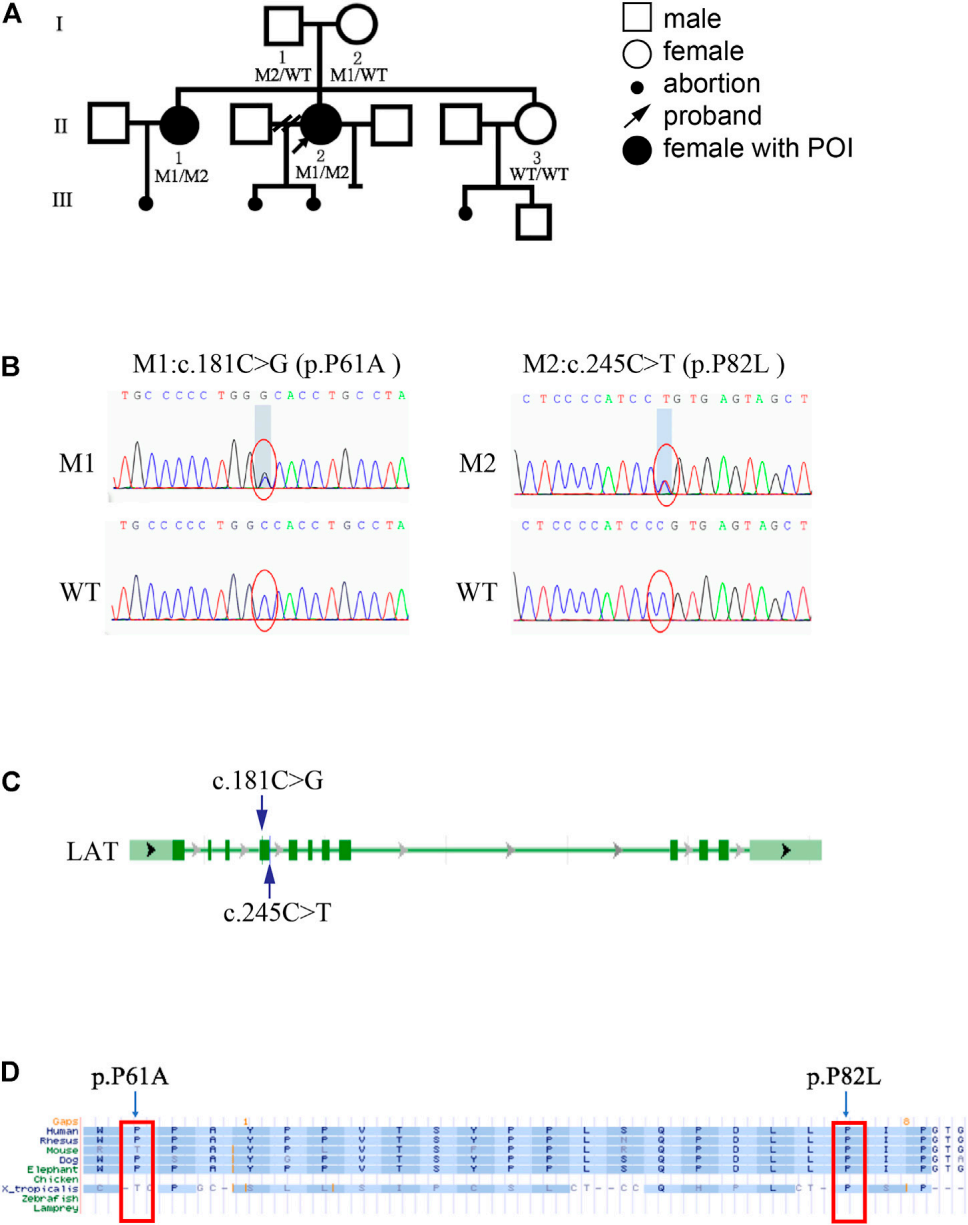
Genetic and bioinformatic analysis of *LAT* mutations **(A)** Family pedigree of the POI family. M1: c.181C > G (p.P61A); M2: c.245C > T (p.P82L). **(B)** Sequencing profiles of the pedigree. **(C)** Schematic representation of the *LAT* gene. The blue arrows indicate the variant positions. **(D)** The *LAT* variants (M1/M2) identified in this study were located at conserved amino acid residues among different species.

The family enrolled in this study comprises two sisters suffered from POI (Figure 1A). Chromosomal abnormalities, FMR1 gene premutation, autoimmune disorders, ovarian surgery history, or chemo-/radiotherapy history was absent in any of the family members, and the mother had regular menses before the age of 50. The proband II-2 presented to our hospital with symptoms of oligomenorrhea and infertility at the age of 29. Hormonal assays revealed high FSH (15.4 IU/L) and low AMH (0.13 ng/ml) plasma levels. She was diagnosed with diminished ovarian reserve. Subsequent test results showed that her FSH level reached 28.3 IU/L, and she was diagnosed with POI at the age of 31. Her sister was clinically diagnosed with POI, reported as FSH 161.18 IU/L, AMH 0.01 ng/ml, and LH 52.3 IU/L at the age of 36.

Whole exome sequencing was carried out in this family to screen for potential mutations. There are 9 mutant genes in all that agree with recessive inheritance model of the POI family, including *LAT, UBXN11, ABCA9, OBSCN, SKOR2, OVOS, FADS6, LILRA6, Clorf98* (Supplementary Table S1). Considering population frequency, we excluded variants with minor allele frequencies >0.01 in the following databases (gnomeAD, inhouse, TGP_CHS, ExAC). Of these, only nonsynonymous variants in exonic and splicing regions which are relevant to ovary function were included. Further filtering by CADD_phred, Polyphen2humvarPred, and SIFTPred yielded single compound heterozygous mutations (c.245C > T and c.181C > G) in *LAT* gene (Figure 1B). Both *LAT* variants were predicted to be deleterious by SIFT, damaging/possibly damaging by Polyphen2, with high CADD score (Table 1). After verifying the outcome of WES via Sanger sequencing, we carried out bioinformatics analysis on the discovered mutant variants. The results showed that these two mutations affected residues that were conserved across different species examined (Figure 1D).

**TABLE 1:** Mutation site information of *LAT* gene.

Variants	M1	M2
cDNA change	c.181C > G	c.245C > T
Protein change	p.P61A	p.P82L
CADD_phred	5.824	28.8
Polyphen2HumVarPred	Possibly damaging	Damaging
SIFTPred	Deleterious	Deleterious

### 3.2 Characterization of Mutant LAT Protein

**FIGURE 2:**
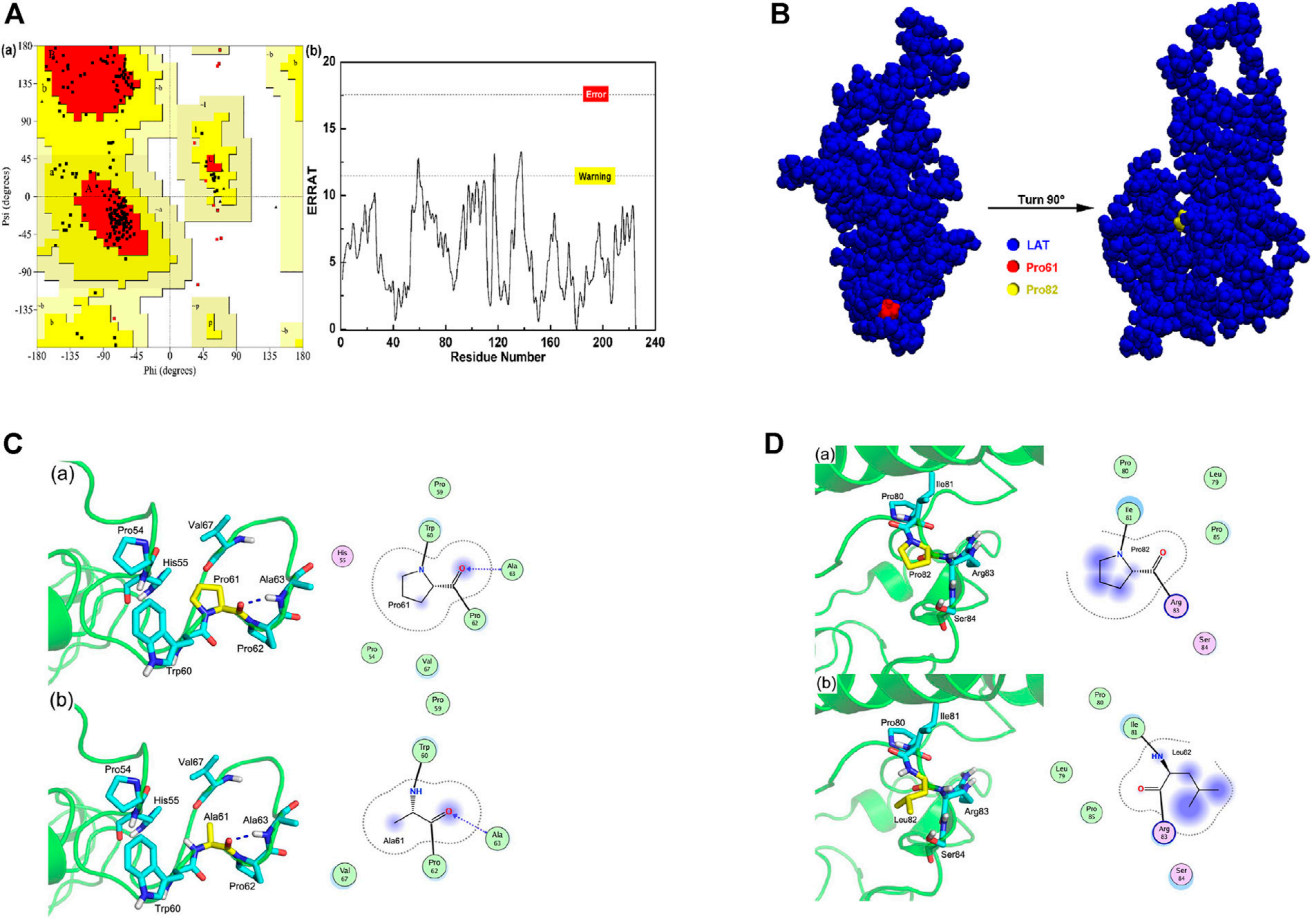
LAT protein structure modeling and mutant prediction **(A)** Ramachandran plot A(a) and ERRAT score A(b) for the LAT protein model. **(B)** LAT protein model and mutation site information. **(C)** Interaction of amino acid 61 with surrounding residues in wild-type LAT protein C(a) and p.P61A mutant C(b). **(D)** Interaction of amino acid 82 with surrounding residues in wild-type LAT protein D(a) and p.P82L mutant D(b).

The three-dimension structure of LAT protein was constructed by Rosetta. As shown by Figure 2A, the Ramachandran plot results for the LAT protein model was 97.4%, and the ERRAT score was 97.75. Based on these results, the constructed LAT structure was seen to be reasonable and could be used as templates for the following research. Figure 2B showed that both mutation sites of the LAT were located inside the LAT protein. Specifically, the P61A and P82L mutants of LAT altered their interactions with surrounding residues, which could change the internal structure of the protein (Figures 2C,D).

### 3.3 Expression of LAT in Ovary and Granulosa Cells

**FIGURE 3:**
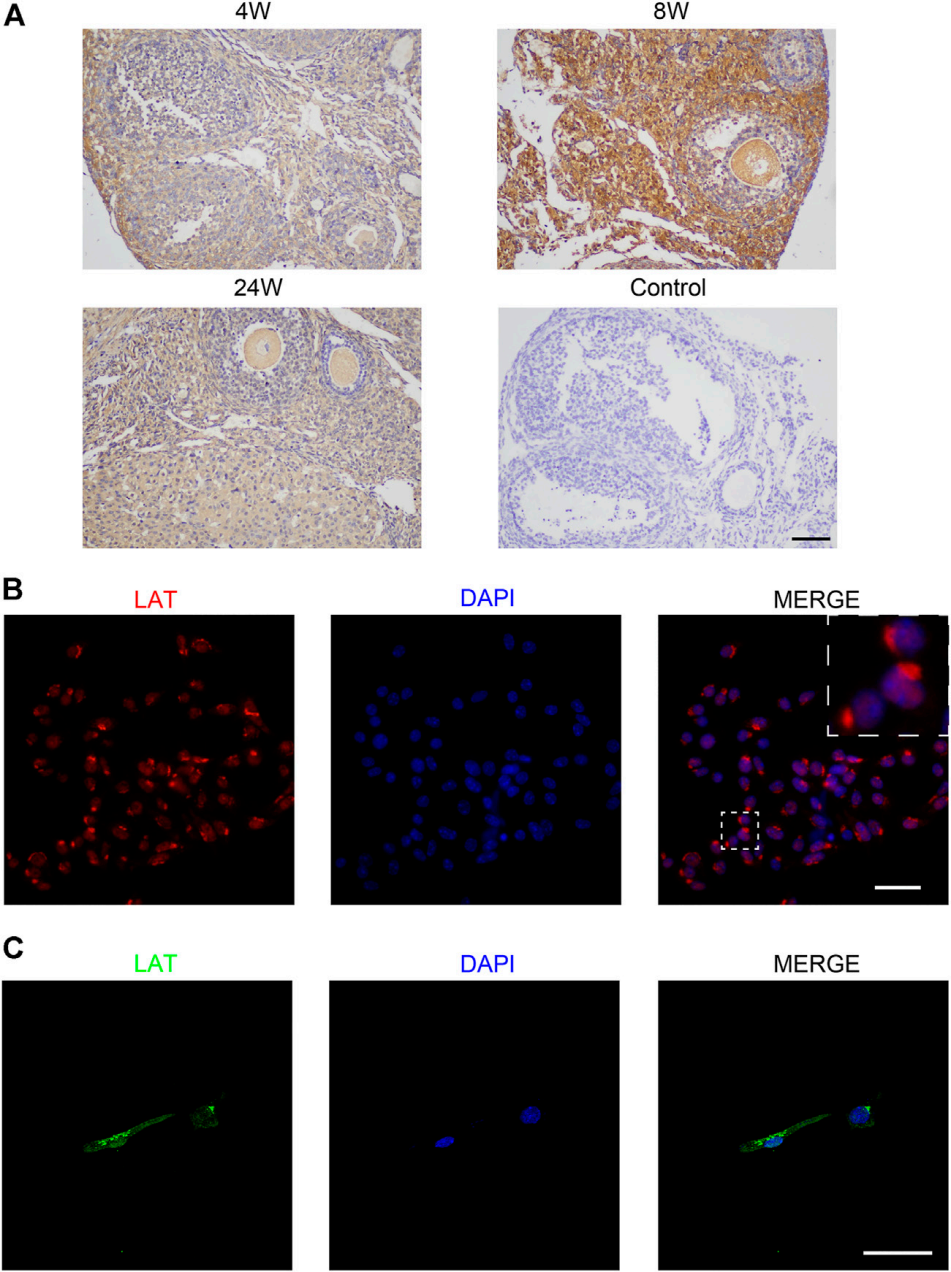
Expression of LAT in ovary and granulosa cell **(A)** Immunohistochemical staining of LAT protein in ovaries of 4 week-old, 8 week-old, and 24 week-old mice. Control was 8 week-old mouse ovary without primary antibody. The scale bar represents 100 μm. **(B)** Immunofluorescent staining of LAT protein in mouse ovarian granulosa cells. The scale bar represents 50 μm. **(C)** Immunofluorescent staining of LAT protein in human ovarian granulosa cells. The scale bar represents 50 μm.

To explore the function of LAT in the ovary, 4 week-old, 8 week-old, and 24 week-old wild-type mice were used to identify the cellular localization and expression dynamics of LAT in mouse ovary. The immunohistochemistry results confirmed that LAT was expressed in mouse ovary. Specifically, LAT protein was detected in GCs of all follicular development stages. We also found that the expression level of LAT was highest in the ovary of 8-week-old mice (Figure 3A), which may underline the importance of LAT protein during the reproductive period. To better visualize the localization and expression pattern of LAT in GCs, immunofluorescent staining was performed in both mouse and human GCs. The results showed that LAT protein was aggregated around the nucleus of both cells (Figures 3B,C).

### 3.4 Effects of *LAT* Pathogenic Variants on LAT Protein Expression

**FIGURE 4:**
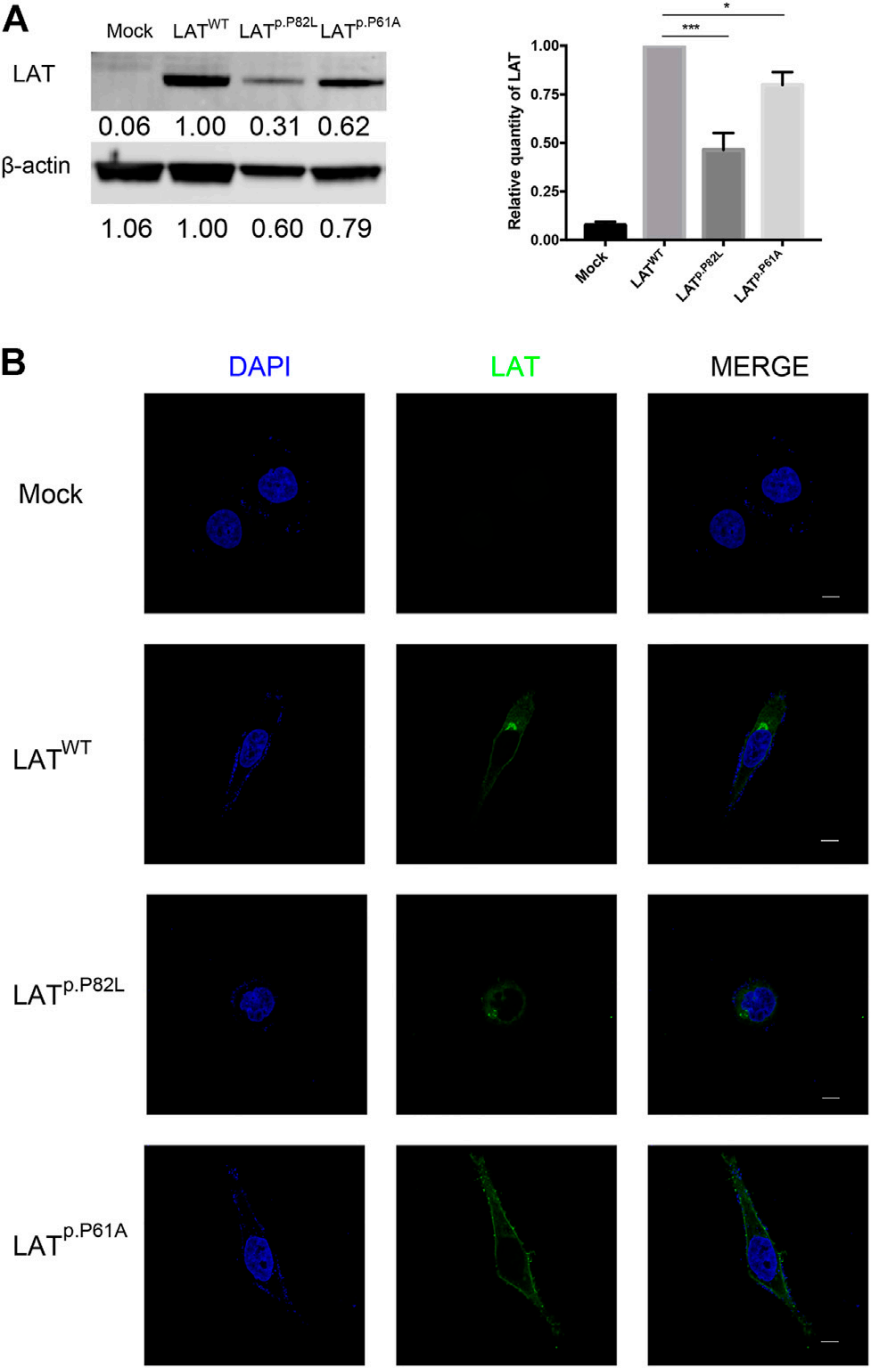
Effects of LAT pathogenic variants on LAT expression **(A)** Western blot analysis of the protein expression levels of wild-type and two altered proteins (P82L and P61A). The densitometric units of altered LAT proteins were normalized to that of the wild-type LAT. *β*-actin was used as the loading control. Values were presented as mean ± SD, N = 3. **(B)** Immunofluorescent staining of LAT protein in LAT^WT^, LAT^p.P82L^, and LAT^p.P61A^ groups. The scale bar represents 10 μm.

To further explore the biological effects of *LAT* pathogenic variants (PVs), equal amounts of wild-type (WT) and mutant (P61A and P82L) expression plasmids were transfected into COV434 cells (a human ovarian granulosa cell-like tumor cell line). QPCR and western blot were performed to check the transfection efficiency. Western blot analysis also revealed that the amounts of LAT protein were significantly downregulated in the mutant groups compared with the wild-type group (Figure 4A), indicating that these two mutation sites affected protein expression levels. Consistently, confocal fluorescent microscopy images of the transfected cells demonstrated decreased concentrations in the mutant groups (Figure 4B). We observed 97 cells in the P61A group. Interestingly, 91 (93.8%) showed that P61A mutation changed the subcellular localization of LAT protein, with LAT protein located in the cell membrane. Of the 99 observed cells in the P82L group, 78 (78.8%) exhibited the typical nucleus aggregated pattern, while others did not, which may due to different cell section as well as the decreased concentration reason.

### 3.5 LAT Pathogenic Variants Suppress Proliferation and Promote Apoptosis of GC

**FIGURE 5:**
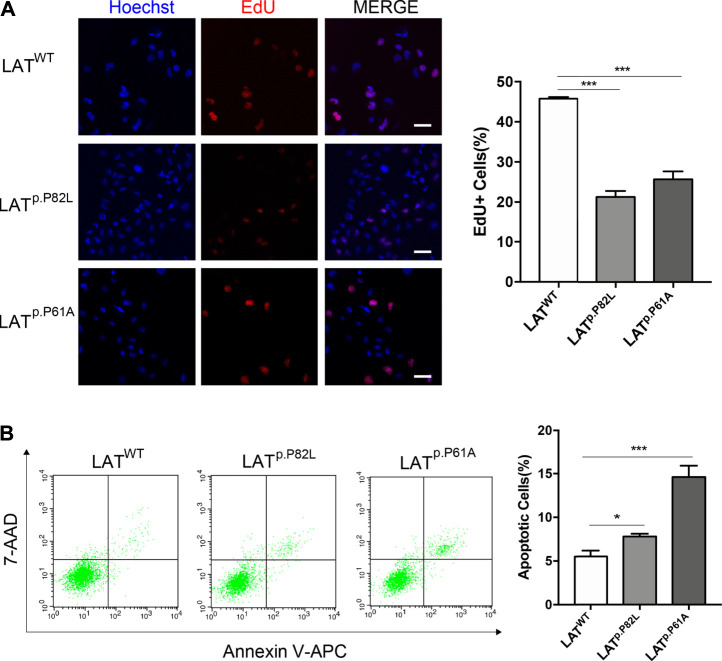
*LAT* pathogenic variants suppress proliferation and promote apoptosis of GC **(A)** EdU staining assay demonstrating the proliferation ability of GCs in LAT^WT^, LAT^p.P82L^, and LAT^p.P61A^ groups. The scale bar represents 50 μm. The percentages of EdU-positive GCs in each group are also presented in a graph next to images. Data are presented as mean ± SD, N = 3. ***p < 0.001. **(B)** Flow cytometric analysis of apoptotic GCs in LAT^WT^, LAT^p.P82L^, and LAT^p.P61A^ groups. The percentages of apoptotic GCs in each group are also presented in a graph next to images. Data are presented as mean ± SD, N = 3. *p < 0.05. ***p < 0.001.

Having observed the reduction of LAT expression caused by P82L and P61A mutants, we further investigate their effects on GCs. EdU staining showed that the percentage of EdU + cells of P82L and P61A group was 21.22 and 25.66% respectively, while that of WT group was 45.75%, indicating P82L and P61A mutants in LAT will reduce GC proliferation ability (Figure 5A). As shown by flow cytometry results, the percentage of apoptotic cells was significantly increased in P82L and P61A group (7.79 & 14.66%) as compared with the WT group (5.50%), indicating a higher apoptosis rate caused by the mutants.

## 4 Discussion

In the current study, we detected novel LAT pathogenic variants in a POI pedigree and reported for the first time that LAT, originally revealed to be expressed in T cells, is present in the GCs of the ovary. Genetic and bioinformatic analysis suggested these two mutations affected conserved residues and could change the internal structure of LAT protein. Moreover, *in vitro* functional assays demonstrated that these two variants reduced LAT expression, suppressed GC proliferation, and promoted GC apoptosis, which may contribute to the etiology of POI.

The linker for the activation of T cells (LAT), one of the most important transmembrane adaptor proteins, is reported to play a crucial role in the development, activation, and maintenance of T cells (Balagopalan et al., 2010). Though LAT has no enzymatic activity, it functions by facilitating the formation of multiprotein signaling complexes (Balagopalan et al., 2010; Wang et al., 2018). LAT-knockout (LAT-KO) mice revealed a total block in thymic maturation, without any mature T lymphocytes (Zhang et al., 1999). The described kindred with a homozygous mutation in *LAT* manifested a progressive combined immunodeficiency and profound immune dysregulation (Keller et al., 2016; Bacchelli et al., 2017). However, no reproductive phenotypes have been described in these syndromic cases with LAT variants. Here, we reported for the first time the clinical course and reproductive findings in a family with compound heterozygous mutations in LAT. Notably, both the patients did not present any immune deficiency or autoimmunity related symptoms. Also, the common immunologic test results were reportedly normal. This may be due to the different functions of one gene performing in different cells. Possibly, P61A and P82L of LAT play an important role in GCs via impacting GC survival, while these two sites are not of vital importance in the T cells.

Previous study has shown that LAT forms two distinct cellular pools, one at the plasma membrane and one in intracellular compartments. The distribution of LAT between these two pools is dependent on intracytoplasmic residues, and LAT recruitment at the right place is essential for its function (Bonello et al., 2004; Carpier et al., 2018). This may explain why P61A mutation changed the subcellular localization of LAT protein, and further influence the cell function. Though no significant change of LAT protein distribution was found in P82L plasmid transfected cells, an obvious reduction in LAT expression was observed, which may account for the difference between WT and P82L plasmid transfected cells.

Granulosa cells, the somatic components of the follicles, play a crucial role in coordinating folliculogenesis. They function via direct communication with the oocytes and theca cells, response to pituitary hormones, as well as their capability to produce necessary nutrients and steroids to the oocytes (Hirshfield, 1991; Georges et al., 2014; Zhang et al., 2014). GC dysfunction is closely related to a great number of ovarian pathologies (Georges et al., 2014). *In vivo* studies have revealed that diminished GCs could induce follicle atresia and finally result in POI (Ono et al., 2014; Wang et al., 2020). Consistently, our results indicated that these two *LAT* PVs suppress proliferation and promote apoptosis of GC, which could be the pathogenesis of POI.

In summary, we detect novel *LAT* pathogenic variants in a POI pedigree and report for the first time that LAT is present and functional in the GCs of the ovary. Our findings not only shed new light on the role of LAT in GCs, but also broaden the spectrum of genetic causes of POI.

## Data Availability

The data presented in the study are deposited in the Sequence Read Archive repository, accession number PRJNA778345.
